# Case Report: Severe rash/desquamation induced by sorafenib in an uHCC patient and its clinical management

**DOI:** 10.3389/fphar.2022.994865

**Published:** 2022-09-13

**Authors:** Yan Lin, Ping-Yu Liu

**Affiliations:** ^1^ School of Basic Medicine and Clinical Pharmacy, China Pharmaceutical University, Nanjing, China; ^2^ Department of Pharmacy, The Second Affiliated Hospital of Nanjing Medical University, Nanjing, China

**Keywords:** sorafenib, severe rash/desquamation, unresectable hepatocellular carcinoma, case report, CTCEA grade 3

## Abstract

**Background:** Sorafenib-related dermatological toxicity is a well-known adverse reaction that can severely affect therapeutic outcomes. Rash/desquamation with its variable manifestations is one of the common clinical presentations. Currently, no standard continuum of care for sorafenib-related rash/desquamation has been established.

**Case summary:** A 75-year-old woman with colorectal cancer who developed unresectable hepatocellular carcinoma (uHCC) received, six years later, sorafenib 400 mg twice daily. She developed a Grade-3 Common Terminology Criteria for Adverse Events (CTCEA) rash and bullae bilaterally on her lower extremities after 2 weeks of sorafenib use. Rash and blisters began to appear on the left calf and then merged as large bullae full of liquid and spread to both lower extremities. The bullae then erupted and skin began to slough off, which affected the patient’s normal daily functioning. To lessen the condition, sorafenib was stopped permanently and dexamethasone intravenous (IV) infusion at 5 mg daily for 3 days and piperacillin/tazobactam were used. The skin dried without exudate or ulcerations after a month.

**Conclusion:** For severe (CTCAE Grade 3 or above) sorafenib-related rash/desquamation, short-term corticosteroid pulse therapy at large doses is usually effective with routine skin care, and antibiotics can be considered if infection is present. Permanent cessation of sorafenib should be considered if severe manifestations such as erythema multiforme (EM) and Steven-Johnson syndrome (SJS) are suspected.

## Introduction

In recent years, the emergence of molecularly targeted agents has reshaped the therapeutic scheme of cancer treatment. These agents not only prolong patient survival but also circumvent some of the systemic toxicities from conventional chemotherapy. Sorafenib is an oral multi-target kinase inhibitor that targets multiple intracellular (c-CRAF, BRAF, and mutant BRAF) and cell surface kinases (KIT, FLT-3, RET, RET/PTC, VEGFR-1, VEGFR-2, VEGFR-3, and PDGFR–ß), which intervenes in tumor growth and angiogenesis (Bayer HealthCare Pharmaceuticals Inc, 2018). Sorafenib has shown satisfactory efficacy with good tolerance for uHCC treatment in several clinical studies (Llovet et al., 2008; Cheng et al., 2009; Lencioni et al., 2012). In 2008, it was approved for uHCC treatment in China. At present, sorafenib remains a first-line systemic treatment options for uHCC, even though promising results with newer regimens are available (National Comprehensive Cancer Network (NCCN), 2022; Vogel and Martinelli, 2021).

Based on the results of sorafenib in uHCC clinical trials, the most commonly reported drug-related adverse effects (AEs) for sorafenib are hand-foot skin reactions (HFSR), diarrhea, fatigue, and rash/desquamation (Llovet et al., 2008; Cheng et al., 2009; Lencioni et al., 2012). With further consideration of its application across different tumor types, sorafenib-related adverse effects that can impact patients’ quality of life and lead to dosage reduction or therapy discontinuation include rash, upper and lower gastrointestinal distress, and fatigue (Llovet et al., 2008; Cheng et al., 2009; Lencioni et al., 2012; Escudier et al., 2009; Gupta-Abramson et al., 2008; Brose et al., 2014). Among these troublesome AEs, rash is prominent and could be profound if early detection and proactive management are missing. Rash often occurs as a popular and erythematous eruption that can involve the extremities as well as the trunk.

Since clinical presentations of sorafenib-related rash may be quite diverse, we report here the case of a patient who developed a systemic rash with severe bullae and diffuse desquamation, which was thought to be sorafenib-related in post-analysis. Severe rash/desquamation from sorafenib has been noted in some clinical trials; however, a complete and elaborate description is usually lacking. There are few sorafenib-related severe rash/desquamation case reports relative to its estimated occurrence rates and large patient population. Furthermore, no standard management protocol for sorafenib-related rash/desquamation is available to facilitate consistent clinical practice. Much more emphasis is needed on promoting the early recognition and scientific management of sorafenib-related rash/desquamation. In this paper, we mainly focus on sorafenib-related rash and desquamation similar to our case: its incidence, common clinical presentations, and possible management approaches.

## Case presentation

The patient was a 75-year-old Asian woman. She had had diabetes mellitus for about 30 years and used insulin, adjusted according to her blood glucose level. She had a laparoscopic cholecystectomy in 2006 and recovered well. In 2015, she was diagnosed with colorectal cancer and had a laparoscopic radical resection of colon cancer on November 9, 2015. She reports no allergies or severe reactions to any medications or substances.

On November 26, 2021, a hepatocellular carcinoma with lymph nodes and peritoneal cavity metastasis was diagnosed based on magnetic resonance imaging and immunohistochemical staining. On December 29, 2021, sorafenib with a full therapeutic dosage of 400 mg twice daily was initiated for her uHCC. Two weeks after initiation, a large blister appeared on her left heel and the patient stopped sorafenib. Blisters then appeared up on her left calf and began to spread to her thigh. About a few days later, a rash and blisters developed on both lower extremities and heels; the blisters grew and merged as bullae. According to the patient, the blisters were bright dusky red with an initial target-like appearance, but then became confluent, bullous, and diffuse. These blisters oozed heavily and erupted, and epidermal sloughing resulted in large, raw, painful areas of denuded skin, making it impossible to wear socks and pants.

The patient suffered from this in bed for a few days before seeking medical attention. Upon ICU admission on January 18 of 2022, the skin had, according to her, improved slightly; unfortunately, the most severe clinical presentation was not captured by the healthcare team. Blisters on both lower limbs gradually burst with obvious exudate. The skin was ulcerated with pus. The estimated affected BSA (body surface area) was 10%. At ICU, the patient had no fever, cough, or headache based on medical records. She was treated as a cutaneous infection with cefazolin 3 g twice daily with daily skin care and was discharged on 27 January 2022. Given its urgent nature and the poor prognosis for the patient, histopathological diagnostic exams were not implemented.

**FIGURE 1:**
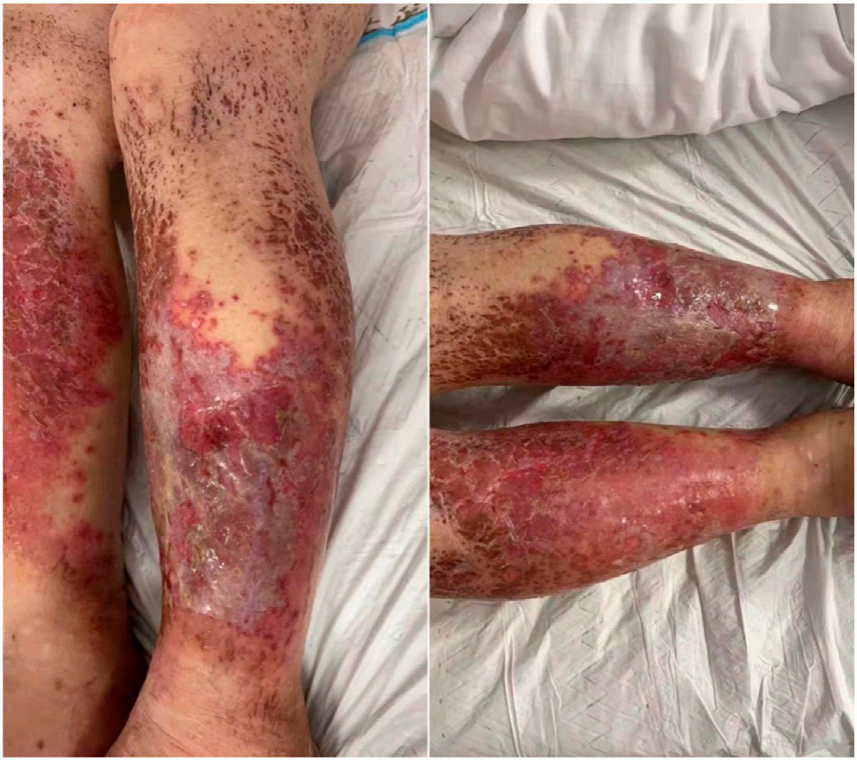
Skin appearance 4 weeks after the onset of skin reaction.

**FIGURE 2:**
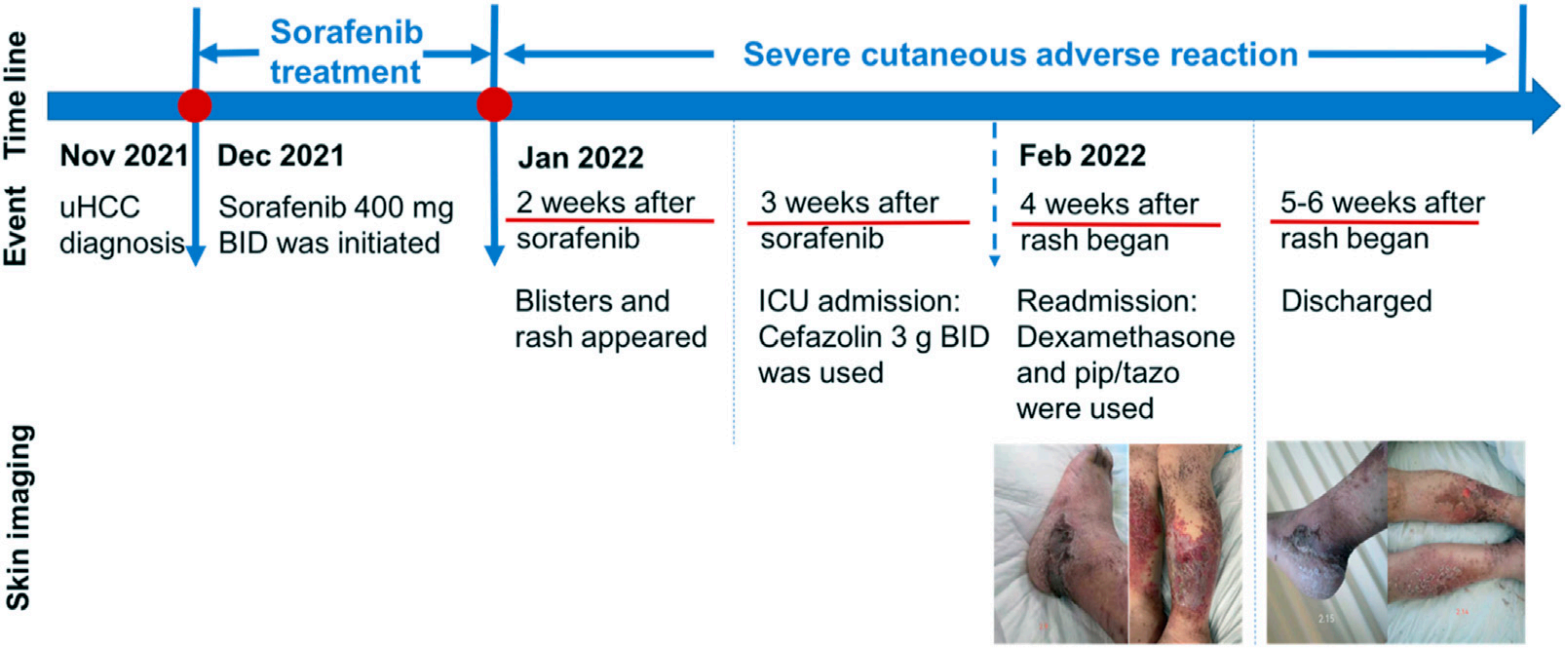
Evolution of clinical events and skin changes. uHCC, unresectable hepatocellular carcinoma; BID, twice daily; pip/tazo, piperacillin/tazobactam.

To seek further evaluation and care, she was readmitted to the Oncology Department on 7 February 2022, approximately 4 weeks after the onset of the skin reaction. Upon readmission, large erythema and bullae along with exudate and pus appeared in front of the tibia, the skin lesions on the thighs had basically crusted, and edema on both legs and feet was obvious, as shown in Figure 1. A complete blood count test on Day 1 revealed several abnormities: increased percentage of neutrophils, decreased percentage of lymphocytes, and decreased percentage of eosinophils. On Day 7, the differential count as well as its percentage was normal (Table 1). During the second hospitalization, she was empirically treated with piperacillin/tazobactam for skin infection, dexamethasone 5 mg IV infusion daily for 3 days, and recombinant bovine basic fibroblast growth factor for external use to promote skin healing. The wound surface gradually dried and the exudate significantly decreased. She was discharged on 17 February 2022 with mild edema and multiple scars without exudate or wound ulceration in both lower extremities. The timeline of this severe cutaneous adverse reaction and partial skin healing process are summarized in Figure 2.

**TABLE 1:** Changes in laboratory results during the second hospitalization.

Items	Units	References range	Results on Day 1	Results on Day 7
WBC	x10^9/L	4–10	6.94	5.25
RBC	x10^12/L	3.5–5.5	3.23	3.08
HGB	g/L	110–150	93	88
HCT	%	37–49	30.40	28.40
MCV	fL	80–100	94.1	92.2
MCH	pg	27–33	28.8	28.6
MCHC	g/L	320–360	306	310
RDW	%	11.5–14.9	17.7	17.6
Platelet count	x10^9/L	100–300	158	177
MPV	fL	6–11.5	10.9	10.4
Differential				
Total neutrophil count	x10^9/L	2–7.5	5.69	3.19
Total lymphocyte count	x10^9/L	0.8–4	0.88	1.32
Total monocyte count	x10^9/L	0–0.8	0.36	0.57
Total eosinophil count	x10^9/L	0–0.8	0.00	0.16
Total basophil count	x10^9/L	0–0.2	0.01	0.01
Neutrophils, %	%	50–75	82.0	60.8
Lymphocytes, %	%	20–40	12.7	25.1
Monocytes, %	%	3–12	5.2	10.9
Eosinophils, %	%	0.5–8	0.0	3.0
Basophils, %	%	0–2	0.1	0.2

WBC, white blood count; RBC, red blood count; HGB, hemoglobin; HCT, hematocrit; MCV, mean corpuscular volume; MCH, mean corpuscular hemoglobin; MCHC, mean corpuscular hemoglobin concentration; RDW, red blood cell distribution width; MPV, mean platelet volume.

## Discussion

### Incidence and clinical presentations of sorafenib-related rash/desquamation

The incidence of sorafenib-related skin rash and desquamation varies by clinical trial, but it is one of the most common dermatological reactions to sorafenib. The severity and incidence of rash/desquamation associated with sorafenib were analyzed based on a meta-analysis that included patients with solid tumors receiving sorafenib single agent with an initial dose of 400 mg twice daily. The incidence of all-grade and high-grade (Grade 3 or above) rash/desquamation were 35.4% (95% CI 0.29–0.43) and 5.0% (95% CI 0.04–0.07) respectively. In addition, the overall risk ratio for all-grade rash/desquamation was 2.73 (95% CI 1.66–4.49) in the sorafenib group compared with the control group, suggesting that sorafenib was associated with a significantly increased risk of rash/desquamation (Zhang et al., 2011). Another article reviewed the results from the phase I to III trials (Treatment Approaches in Renal Cancer Global Evaluation Trial (TARGETs)) involving patients with metastatic clear-cell renal cell carcinoma receiving single-agent sorafenib: the incidence of any-grade rash/desquamation was 40%. Some severe presentations included disseminated exanthematous rash and lesions resembling erythema multiforme (EM) (Robert et al., 2009).

Among reported sorafenib-related rash/desquamation, skin reaction generally showed up within the first 6 weeks of sorafenib use (Robert et al., 2009; Zhang et al., 2011; Brose et al., 2014). However, delayed onset was seen in a patient with HCC, in which an itchy rash presented on the trunk nine months after sorafenib initiation (Sarkodie and Ross, 2010). Manifestations of sorafenib-related rash/desquamation are dramatically diverse. They could present as eczema (Sarkodie and Ross, 2010; Bauer et al., 2008), EM (Kodaira et al., 2010; Ikeda et al., 2012; Sohn et al., 2015), generalized maculopapular eruptions (Sohn et al., 2015), and SJS (Sohn et al., 2015; Ikeda et al., 2013).

This article presents a case of severe rash after 2 weeks of sorafenib administration. Unlike other cases, the major characteristic of our case is a large, confluent, and diffuse rash with bullae and epidermal sloughing. Its onset is consistent with the general onset timing of sorafenib-related rash/desquamation, as discussed above. In addition, the patient was taking no medications at the time except for insulin, and no other contributing factors could explain this. Insulin hypersensitivity was quickly ruled out because our patient had been using it for many years without any allergic reactions. Hence, sorafenib was the culprit, which was agreed upon across the healthcare team. A more definitive diagnosis was then needed after sorafenib-related rash and desquamation were identified.

Since skin biopsy or histopathological testing was not available, a definitive diagnosis cannot be confirmed. There were a few signs that might indicate a diagnosis of sorafenib-induced SJS. Her presence of epidermal detachment and onset timing were compatible with SJS characteristics; however, mucous membrane involvement and prodrome with systemic symptoms, which are usually seen in classic SJS cases, were lacking in our case (Roujeau and Stern, 1994). Of course, these neither confirm nor exclude the diagnosis of SJS. Again, delayed medical intervention and the absence of skin biopsy make diagnosis obscure.

ALDEN (algorithm of drug causality for epidermal necrolysis (EN)) is an algorithm developed to assess the individual causality of drug-induced SJS or toxic epidermal necrolysis (TEN). It is especially helpful for patients who take multiple medications, each of which will have a score ranging from −12 to +10 calculated on six parameters (Sassolas et al., 2010). The higher the score, the higher the causality of the drug causing SJS or TEN. The results of this algorithm have been validated as correlating well with those of the European Study of Severe Cutaneous Adverse Reactions (EuroSCAR-study) case-control analysis for drugs associated with EN (*r* = 0.90, *p* < 0.0001); it is an invaluable and sensitive adjunct tool for healthcare providers who focus on pharmacovigilance. Therefore, it was adopted to help analysis of our patient. In our case, the ALDEN score is +4, which suggests a probable causality. Notably, it only indicates a probability, which means further professional judgment, combined with clinical findings and skin biopsy, is required to confirm the diagnosis.

Even though the diagnosis of sorafenib-induced SJS in our case is inconclusive, its severity deserves our attention and vigilance. Based on the newest version of CTCAE grading (National Cancer Institute, 2017), our patient was rated as Grade 3 “Rash maculo-papular” or “Bullous dermatitis”, which requires affected lesion coverage of >30% BSA with moderate or severe symptoms and limited self-care ADL (activity of daily living). Although the percentage of BSA affected was not quite accurate because she did not present at the most severe time, her situation would still be Grade 3 based on the category of “Skin and subcutaneous tissue disorders—other, specify”. Her encounter certainly required hospitalization and was of medical significance, leading to the permanent withdrawal of sorafenib. Because of this event, she was overwhelmed, had a negative attitude toward further treatment, and only accepted symptomatic management for her cancer.

Due to the poor prognosis of our patient, it was almost impossible to proceed with skin biopsy or genetic testing, which was the main limitation in our case. However, some human leukocyte antigens (HLAs) were known to be linked with cutaneous adverse reactions from drugs. For sorafenib, HLA-B*46:01, HLA-A*24:02, and HLA-Cw*04:01 have been shared in Korean patients who developed cutaneous hypersensitivity from sorafenib (Sohn et al., 2015). For future studies, HLA information needs to be collected and analyzed from a large number of qualified patients affected by sorafenib to predict potential genetic predisposition.

### Management of sorafenib-related rash/desquamation

The management of sorafenib-related rash/desquamation depends largely on presentation and severity. In most clinical trials and reports, common approaches include symptomatic management, sorafenib dose adjustment, or permanent discontinuation . If cutaneous infection is present, antibiotics could be used. Topical therapies (Kodaira et al., 2010; Ikeda et al., 2013), antihistamines (Bauer et al., 2008), and systemic corticosteroids (Kodaira et al., 2010; Sohn et al., 2015; Ikeda et al., 2013) have also been used in some cases.

Systemic corticosteroids have been used frequently and effectively for drug-induced hypersensitivity management; however, there is no consensus on the choice and administration regimen of individual steroids for sorafenib-related rash/desquamation treatment. Prednisolone (Kodaira et al., 2010; Sohn et al., 2015; Ikeda et al., 2013) or methylprednisolone (Bauer et al., 2008) can be selected; the prednisolone-equivalent dose used in previously reported cases can range from 10 mg/day to 30 mg/day. Moreover, sorafenib-induced EM can disappear within days of sorafenib discontinuation in the absence of steroid treatment (Kodaira et al., 2010; Sohn et al., 2015).

Furthermore, tolerance induction trials have been successful in situations where sorafenib is urgently needed and cannot be withdrawn. In an oral tolerance induction trial, a patient is pre-medicated with an oral corticosteroid and as-needed antihistamine before consecutive assigned doses of sorafenib with intervals were given (Bauer et al., 2008). Tolerance induction may be considered when the full therapeutic dose of sorafenib needs to be re-achieved after the first occurrence of rash; however, this approach may warrant more intensive care and monitoring.

With proper care and treatment, rash/desquamation usually disappears within 2 weeks of sorafenib discontinuation (Kodaira et al., 2010), even in sorafenib-induced SJS (Sohn et al., 2015; Ikeda et al., 2013). After the first occurrence of sorafenib-related rash/desquamation, re-commencing sorafenib should be done extremely cautiously. In some cases, it may be prudent to restart sorafenib with dose modification, although, in some cases, re-challenge could lead to a recurrence of rash (Kodaira et al., 2010; Sohn et al., 2015). If EM or SJS is suspected, re-administration of sorafenib is not recommended as EM and SJS are type IV hypersensitivity reactions.

In our case, because of its severity and the possibility of SJS, sorafenib was permanently discontinued. Dexamethasone was used for 3 days, with an equivalent dose of prednisolone of 31 mg. Short-term steroid pulse therapy with large dosage is uncommon in managing sorafenib-induced rash/desquamation. There was one case in which prednisolone 30 mg/day for 2 days was adopted to manage generalized maculopapular eruptions caused by a 10-day treatment of sorafenib (Sohn et al., 2015). In previously reported cases, oral administration of low-dose steroids (10–15 mg of prednisolone equivalent dose) was more common. As reported, upon sorafenib discontinuation and steroid treatment, cutaneous symptoms usually disappear within 2 weeks. Our patient recovered a week after dexamethasone use and sorafenib withdrawal.

There is, so far, no consensus on the steroid dose intensity and treatment duration for severe cutaneous adverse reactions caused by sorafenib, nor for multi-target kinase inhibitors. A corticosteroid regimen in our case may not be generalized to every case but provides an alternative for similar cases. From this case, we realized the urgency of giving full attention to sorafenib-related rash/desquamation. Proactive management, early recognition, and effective patient communication are the areas where more efforts are needed to eliminate severe adverse reactions (Walko and Grande, 2014). In addition, one important future direction is to establish a scientific pharmacovigilance plan to guarantee the effectiveness and safety of sorafenib use.

## Conclusion

Our case describes a severe cutaneous adverse reaction after 2 weeks of sorafenib therapy which affected the patient’s ADL. Compared to previously reported cases, diffuse rash and desquamation of both lower limbs are the main features of our case, and dexamethasone IV infusion 5 mg for 3 days plus antibiotic administration could be an effective approach for alleviating symptoms. Because of the lack of skin biopsy, we decided to not investigate the accuracy of the diagnosis. Our case implies that sorafenib-related rash/desquamation could be detrimental, impacting a patient’s daily life and normal function if left untreated; therefore, prompt diagnosis with a thorough investigation is warranted for proper treatment. If EM or SJS is highly suspected, permanent cessation of sorafenib should be implemented. Given the variable presentations of sorafenib-related rash/desquamation, the medical team should remain vigilant at all times. In addition, with the increasingly broad use of sorafenib in diverse tumor types, the early screening and detection of dermatological toxicity as well as appropriate management and routine follow-up should be in place.

In summary, our case adds to the accumulating evidence that corticosteroids are effective in managing severe cutaneous adverse reactions induced by sorafenib, but that dose intensity and treatment duration must be further tested. At the same time, there is an urgent need for strong evidence-based clinical treatment guidelines for the management of sorafenib-related rash/desquamation. It is increasingly important to realize that optimizing quality of life and prolonging survival are equally important in cancer treatment, especially for advanced cancer patients with a limited life span. Minimizing adverse reactions and toxicities from medication therapy is the cornerstone to maximizing therapeutic outcomes and patients’ quality of life.

## Data Availability

The original contributions presented in the study are included in the article/Supplementary material, and further inquiries can be directed to the corresponding author.
